# Detection of Clenbuterol-Induced Changes in Heart Rate Using At-Home Recorded Smartwatch Data: Randomized Controlled Trial

**DOI:** 10.2196/31890

**Published:** 2021-12-30

**Authors:** Willem O Elzinga, Samantha Prins, Laura G J M Borghans, Pim Gal, Gabriel A Vargas, Geert J Groeneveld, Robert J Doll

**Affiliations:** 1 Centre for Human Drug Research Leiden Netherlands; 2 Leiden University Medical Center Leiden Netherlands; 3 CuraSen Therapeutics Inc San Carlos, CA United States

**Keywords:** photoplethysmography, smartwatch, wearable, at-home, heart rate, RCT, wearable device, digital health, cardiovascular, cardiology, sensors, heart rate sensor, smart technology

## Abstract

**Background:**

Although electrocardiography is the gold standard for heart rate (HR) recording in clinical trials, the increasing availability of smartwatch-based HR monitors opens up possibilities for drug development studies. Smartwatches allow for inexpensive, unobtrusive, and continuous HR estimation for potential detection of treatment effects outside the clinic, during daily life.

**Objective:**

The aim of this study is to evaluate the repeatability and sensitivity of smartwatch-based HR estimates collected during a randomized clinical trial.

**Methods:**

The data were collected as part of a multiple-dose, investigator-blinded, randomized, placebo-controlled, parallel-group study of 12 patients with Parkinson disease. After a 6-day baseline period, 4 and 8 patients were treated for 7 days with an ascending dose of placebo and clenbuterol, respectively. Throughout the study, the smartwatch provided HR and sleep state estimates. The HR estimates were quantified as the 2.5th, 50th, and 97.5th percentiles within awake and asleep segments. Linear mixed models were used to calculate the following: (1) the intraclass correlation coefficient (ICC) of estimated sleep durations, (2) the ICC and minimum detectable effect (MDE) of the HR estimates, and (3) the effect sizes of the HR estimates.

**Results:**

Sleep duration was moderately repeatable (ICC=0.64) and was not significantly affected by study day (*P*=.83), clenbuterol (*P*=.43), and study day by clenbuterol (*P*=.73). Clenbuterol-induced changes were detected in the asleep HR as of the first night (+3.79 beats per minute [bpm], *P*=.04) and in the awake HR as of the third day (+8.79 bpm, *P*=.001). The median HR while asleep had the highest repeatability (ICC=0.70). The MDE (N=12) was found to be smaller when patients were asleep (6.8 bpm to 11.7 bpm) than while awake (10.7 bpm to 22.1 bpm). Overall, the effect sizes for clenbuterol-induced changes were higher while asleep (0.49 to 2.75) than while awake (0.08 to 1.94).

**Conclusions:**

We demonstrated the feasibility of using smartwatch-based HR estimates to detect clenbuterol-induced changes during clinical trials. The asleep HR estimates were most repeatable and sensitive to treatment effects. We conclude that smartwatch-based HR estimates obtained during daily living in a clinical trial can be used to detect and track treatment effects.

**Trial Registration:**

Netherlands Trials Register NL8002; https://www.trialregister.nl/trial/8002

## Introduction

In early phase clinical trials, heart rhythm is one of the vital signs that is routinely measured with laboratory grade equipment (eg, electrocardiogram [ECG]) for safety and to detect adverse treatment effects [1]. Repeated recording of heart rhythm parameters, such as the heart rate (HR), provides clinically relevant information on the effects of a novel drug intervention. The monitoring of the HR aids in the detection of early signs of distress [2], for example, pharmacologically induced arrhythmias, hypotension, or gastrointestinal symptoms. Currently, there is a trend towards at-home observation of patients enrolled in clinical trials [3]. Recording of the HR in a home environment would require a system that is capable of continuous recording and is relatively user-friendly.

One of the strategies to obtain at-home recordings of the HR is to use a Holter monitor. This requires a setup in which a small device and several electrodes are attached to a patient, which could make this device cumbersome to use. Various smaller wearable devices are now widely available which include ECG patches (eg, [4,5]) or photoplethysmography (PPG) sensors integrated in wrist-worn devices [5]. While continuous monitoring using an ECG patch can be accurate and sensitive to medication (eg, [6]), wrist-worn PPG sensors are more convenient to use. Additionally, wrist-worn HR monitors using PPG sensors are relatively cheap commercial devices and may provide clinically relevant information in addition to being user-friendly. Furthermore, PPG HR estimates have shown to correlate well with ECG HR estimates in healthy participants [7,8]. The PPG sensor measures volumetric changes in blood flow within the microvasculature during the cardiac cycle [9]. In clinical trials, such monitors could be used to unobtrusively obtain estimates of the HR at home (ie, away from the clinic) and during daily life activities (eg, while asleep or awake). Therefore, if HR monitoring for a longer duration is required, smartwatch HR monitors could potentially result in shorter or fewer clinic visits, reducing the total trial costs.

Before using such a device as a biomarker source in clinical trials, the biomarker correlation with the actual HR, its repeatability, and its sensitivity need to be evaluated. HR estimates from PPG wearables have been demonstrated to correlate well with the actual HR [7,8]. Next, biomarkers must be able to produce repeatable values in the presence of measurement noise within the subject. Finally, the sensitivity of the biomarker to detect meaningful changes must be demonstrated. Before these qualities can be assessed, however, the continuous stream of HR estimates must be preprocessed into 1 or several biomarkers.

HR estimates obtained from a wearable device during physical activity may show higher variability and are less accurate than estimates obtained while at rest [10]. As a result, it is useful to distinguish between the active and rest HR estimates prior to further analysis. A relatively simple method to distinguish between rest and active estimates is by splitting the HR estimates into two groups: HR estimates while asleep and HR estimates while awake. As an alternative to asking patients to keep track of their sleep pattern by means of a diary, wearable devices can also provide information on sleep patterns (eg, sleep duration, light or deep sleep). However, while the estimated sleep duration can be considered reasonably reliable, estimates of the substates (eg, light or deep sleep) cannot [11]. Therefore, the estimated sleep start and end times can be used to split the HR stream into awake and asleep estimates.

Here, we evaluate the feasibility of using a smartwatch-based HR monitor in a clinical trial. We first evaluate the repeatability of the estimated sleep durations. Then, after evaluating the repeatability of smartwatch-obtained HR over a time period of 1 week, we present the sensitivity of the smartwatch to detect drug-induced changes in the HR. We used smartwatch-based HR monitoring in a trial of patients with Parkinson disease (PD) who were treated with clenbuterol, a β_2_-adrenoceptor agonist, in a placebo-controlled fashion. The work presented here was part of a trial aimed at demonstrating the central nervous system effects of the drug. The results of that trial will be reported separately.

## Methods

### Study Location and Ethical Approval

The work presented here is a part of an exploratory objective of a larger clinical trial. The trial was approved by the Stichting Beoordeling Ethiek Biomedisch Onderzoek ethics committee, Assen, the Netherlands, and was executed in accordance with the Declaration of Helsinki at the Centre for Human Drug Research, Leiden, the Netherlands. The trial is registered in the Netherlands Trial Register under NL8002. Here, we report the exploratory objective of evaluating the effects of clenbuterol, a direct-acting sympathomimetic with predominant β_2_-adrenoceptor selectivity, on HR measured with a smartwatch.

### Participants

All participants provided written informed consent. A total of 12 patients with PD (10 men, 2 women; mean age 64.3 years, SD 6.6 years) participated in the trial. The trial included patients at a Hoehn and Yahr stage between 1 and 3 and a Mini Mental Status Examination score of above 25. Patients taking β-blockers or β-agonists for reasons other than for treatment of tremor were not eligible. Other medication for PD (eg, levodopa, carbidopa, and benserazide) had to be at a stable dosage for at least 3 months. Patients were otherwise healthy or stable (eg, stable hypertension for at least 3 months, stable dyslipidemia, normal or not clinically significant 12-lead ECG, normal laboratory values including hematology and chemistry values). The use of vitamin E (up to 400 IU daily), estrogens, aspirin (81 to 300 mg daily), blood pressure medications (except for adrenergic agents), and cholesterol-lowering agents was allowed if treatment was stable for 3 months prior to screening.

### Study Design

This was a multiple-dose, investigator-blinded, randomized, placebo-controlled, parallel group study. The study duration was approximately 7 weeks, including a screening period of up to 31 days, but a minimum of 6 days (day –6 to day 0), a treatment period of 1 week (day 0 to day 6), and a 1-week follow-up period. Patients started wearing the smartwatch after the screening session.

Patients were randomly assigned to either the placebo or clenbuterol treatment group with a 1:2 ratio (ie, 4 patients were assigned to the placebo group and 8 patients were assigned to the clenbuterol group). Patients continued taking their own medication throughout the full study period. The clenbuterol dose was titrated from 20 µg on the first treatment day (ie, day 0) to 40 µg on day 1 and 80 µg on days 2 through 6. Patients were dosed with either clenbuterol or a matching number of tablets of placebo. Since the tablets of clenbuterol and placebo were nonmatching, patients were instructed not to discuss the tablet appearance with other patients or study site staff.

### Measurements

Measurements were collected using a smartwatch (Steel HR, Withings). During the screening session, patients were given a smartwatch and the Withings Health Mate app was installed on their phones. The app was configured to work with the smartwatch and connected to an account that was used to upload the measurements during the study days. Patients wore the smartwatch at least 6 days prior to the treatment period (ie, day –6 to day 0) and continued wearing the smartwatch during the treatment period (ie, day 0 to day 6). As the quality of the measurements is potentially variable [10], participants were instructed to position and strap the watch such that motion on the skin was minimized throughout the study period.

Data collected by the smartwatch include estimates of the HR and sleep states. The smartwatch provides an HR estimate every 10 minutes. However, when physical activity (eg, running) is detected, the sample frequency increases. The smartwatch provided 3 discrete states for sleep: awake, light sleep, or deep sleep. Only changes in states were stored by the smartwatch. All HR samples and changes in sleep state samples contained timestamps.

### Smartwatch Data Preprocessing

**Figure 1:**
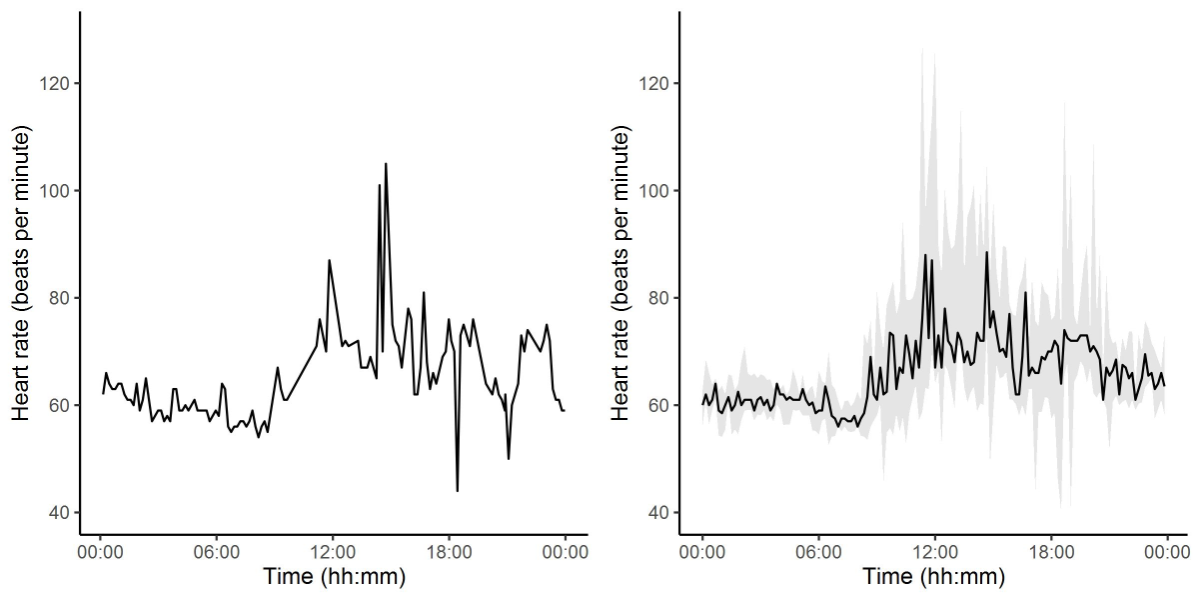
(A) Smartwatch-obtained heart rate (HR) throughout a single day in a single subject. (B) Median heart rate throughout the day for a single subject, with shading between the 2.5th and 97.5th HR percentiles.

The smartwatch continuously captured HR for 1 week prior to treatment and 1 week after treatment (ie, day –6 to day 6). An example HR time series of 1 patient is presented in Figure 1. The left and right panel present the HR throughout a single day and the median HR throughout the baseline period, with the shading representing the 2.5th and 97.5th HR percentiles, respectively.

For further processing steps, we distinguished between the HR while asleep and the HR while awake. The HR while asleep is defined as all HR samples collected between the first moment the smartwatch indicated a sleep state until the last sleep state change of the following day. The HR while awake is defined as all HR samples collected outside the asleep boundaries. Asleep HR series shorter than 3 hours and nights without any detected sleep state were excluded from further analysis. For days with missing sleep states, the awake period was assumed to start at 9 AM and end at 9 PM. For each asleep or awake period and for each patient, HR series were summarized by their 2.5th, 50th, and 97.5th percentile values. From here on, these markers will be referred to as Awake-Low, Awake-Median, Awake-High, Asleep-Low, Asleep-Median, and Asleep-High.

### Statistical Analysis

All data preparation and analyses were performed using the R software package (R Foundation for Statistical Computing). The linear mixed models (LMM) described below were fitted using the nlme package. Restricted maximum likelihood was used for fitting and the Kenward-Roger method was used for estimating the degrees of freedom. Contrasts between treatment days and effect sizes were estimated using the emmeans package. Statistical significance was defined as *P*<.05.

### Repeatability of Smartwatch Sleep Duration Estimates

As HR series might be more variable while asleep than while awake (as found in [10]), the HR series were segmented into asleep and awake periods using the smartwatch-provided sleep states. To assess the reliability of patients’ sleep states, an LMM was fit to estimate if treatment affected the total sleep duration. The intercept, day (ie, day –6 to 6), treatment (ie, placebo or clenbuterol), as well as the interaction between day and treatment were included as fixed effects. Between-subject random effects were only included for the intercept. Type III *F* statistics were used to assess if treatment significantly explained the sleep duration variability. The reliability of sleep duration was quantified as the intraclass correlation coefficient (ICC), which is defined as the ratio between the between-subject variance and the sum of the between-subject and within-subject variance.

### Repeatability and Minimum Detectable Effect of Smartwatch HR Estimates

To assess the repeatability and the minimum detectable effect (MDE) of HR estimates of the markers, only the predose data were considered. An LMM was fitted for each of the 6 markers. The intercept and day (ie, day –6 to day 0) were included as fixed effects. Between-subject random effects were only included for the intercept. The type III *F* statistic of the fixed effect for day was used to assess if structural day-to-day variability was present. The repeatability of each marker was quantified as the ICC.

The MDE values were calculated using an inverse sample size (n=12) calculation. For each marker, the total SD was calculated by taking the square root of the sum of within-subject and between-subject variation. The SD was then used in the inverse sample size calculation together with quantiles from the *t*-distribution with n–1 degrees of freedom for a false positive probability α of .05 and a power of .80.

### Effect of Clenbuterol on the Smartwatch HR Estimates

To estimate the effect of the treatment on the smartwatch-obtained HR, an LMM was fit for each of the smartwatch markers. The intercept, day (ie, day –6 to 6), treatment (ie, placebo or clenbuterol), as well as the interaction between day and treatment were included as fixed effects. Between-subject random effects were only included for the intercept. Type III *F* statistics were used to assess the main and interaction effects. The estimated effects on each dosing day (day 0 to day 6) were contrasted to the average predose (day –6 to day –1) values. Bonferroni correction was applied to correct for multiple testing. The Cohen *d* effect size was calculated as the contrast between HR estimates after clenbuterol treatment and the averaged predose HR, divided by the square root of the sum of the between-subject and within-subject variation.

## Results

All participants completed the experiment procedures. None of the patients were taking β-blockers or β-agonists. There were, on average, 116 awake and 39 asleep HR estimates available per patient per day. Due to the late inclusion of 1 patient, 5 days of baseline data were missing for this patient. Another patient reported sleepless nights due to shoulder pain, resulting in 10 missing nights for this patient. Another total of 14 nights were missing for the other patients due to missing sleep states (n=12) or nights lasting shorter than 3 hours (n=2). For the patients in the clenbuterol group, 4 out of 44 nights were missing prior to dosing and 6 out of 48 nights were missing post dosing.

### Repeatability of Smartwatch Sleep Duration Estimates

The day by treatment interaction effect did not have a significant effect on the total sleep duration (*F*_12,92.5_=0.72, *P*=.73). The sleep duration was also not significantly affected by the main effects day (*F*_12,92.5_=0.60, *P*=.83) and treatment (*F*_1,9.2_=0.69, *P*=.43). The repeatability of the sleep duration was moderate (ICC=0.64).

### Repeatability and MDE of Smartwatch HR Estimates

For all 6 repeatability models, no significant effect of day was detected on the smartwatch HR (Table 1). The repeatability values are presented in Table 2. Except for the Awake-High HR (ICC=0.23), all markers showed moderate repeatability values with ICC values ranging between 0.58 and 0.70. The Asleep-Median marker showed the best repeatability (ICC=0.70). The MDE values (both absolute and relative) are presented in Table 2. The asleep HR markers had lower MDE values than the awake HR markers. The Asleep-Low and Awake-High markers had the best and worst MDE values, respectively.

**Table 1:** Type III *F* statistics for each of the 6 models used to assess the repeatability of the smartwatch HR (heart rate). Each row depicts the values for the day effect.

Model		Degrees of freedom, numerator/denominator	*F* statistic	*P* value
**Awake**				
	Low	5/50.5	1.44	.23
	Median	5/50.4	0.86	.52
	High	5/50.8	0.28	.92
**Asleep**				
	Low	5/40.9	1.93	.11
	Median	5/40.7	1.25	.30
	High	5/40.8	1.14	.35

**Table 2:** The estimated mean predose heart rate (HR), standard error (SE), intraclass correlation coefficient (ICC), and minimum detectable effect (MDE) for each heart rate (HR) marker. The MDE percentage is relative to the estimated mean HR.

HR marker		Mean (SE), bpm^a^	ICC	MDE, bpm (%)
**Asleep**				
	Low	57.6 (1.70)	0.64	6.84 (11.7)
	Median	63.2 (1.36)	0.70	7.79 (12.2)
	High	74.2 (1.94)	0.67	11.71 (15.1)
**Awake**				
	Low	58.2 (2.03)	0.58	10.72 (18.3)
	Median	75.4 (1.56)	0.65	11.45 (15.5)
	High	104.1 (2.55)	0.23	22.13 (21.5)

^a^bpm: beats per minute.

### Effect of Clenbuterol on Smartwatch HR Estimates

**Figure 2:**
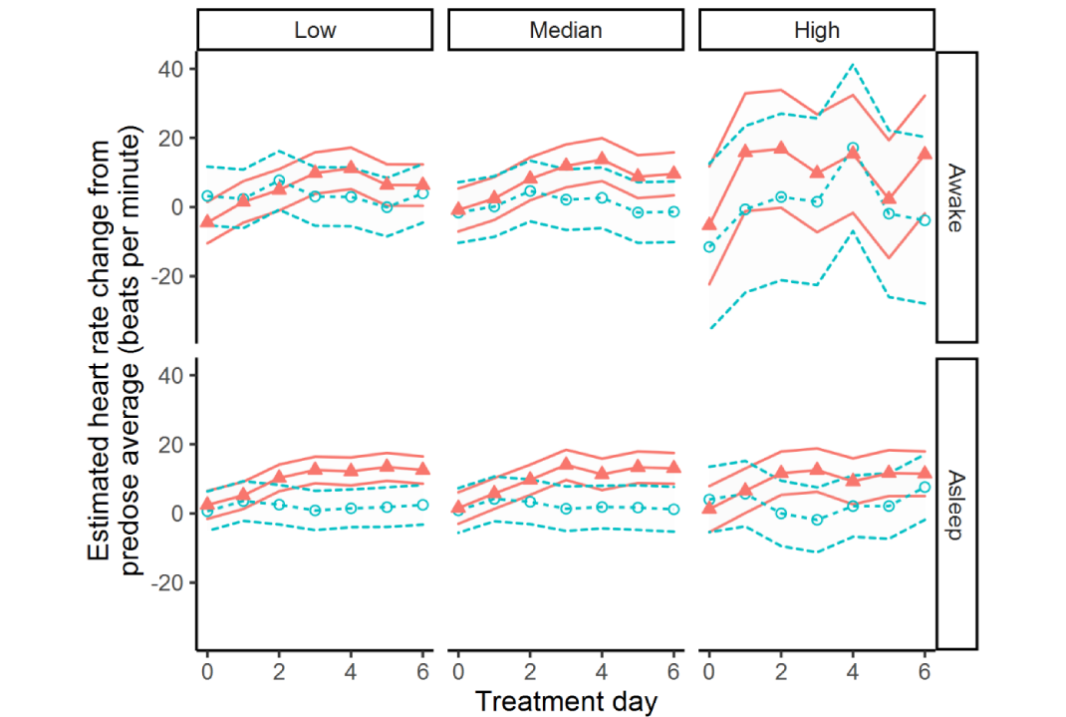
Estimated daily heart rate (HR) changes from average baseline values. The blue circles and orange triangles indicate the HR in the placebo arm and the clenbuterol arm, respectively. The 95% confidence intervals of the estimated HR are indicated by the lines with treatment arm color and style.

Table 3 presents the results of the 6 models used to assess the effect of clenbuterol on each of the smartwatch HR markers. The day by treatment effect is significant for all markers, except for the Awake-High marker. Post hoc comparisons between the average baseline HR and HR during treatment days are presented in Table 4 and in Figure 2. Note that the Awake-High values are not displayed in Table 4 as the day by treatment interaction term was not significant for this marker. No changes in HR were detected for any of the markers in the placebo group. In the clenbuterol group, a change in HR was detected by the Asleep-Low HR marker as of the first night after treatment (+3.79 beats per minute [bpm], *P*=.04). A change in HR was detected by the other asleep HR markers as of the second night (Asleep-Median: +7.9 bpm, *P*<.001; Asleep-High: +9.5 bpm, *P*<.001). For the Awake HR markers, a change in HR was detected by the Awake-Low HR marker as of the third day after treatment (+8.79 bpm, *P*=.001). Table 5 presents the effect sizes to detect changes induced by clenbuterol. The effect sizes are generally better for the asleep HR markers than for the awake HR markers. Additionally, effect sizes increase with increasing dosages of clenbuterol.

**Table 3:** Type III *F* statistics for each of 6 models used to assess the treatment effect on the smartwatch-obtained heart rate.

Model			Low				Median				High		
			*F* (df1/df2)^a^		*P* value		*F* (df1/df2)	*P* value		*F* (df1/df2)			*P* value
**Awake**													
	Day	3.05 (12/115.0)		.001		5.07 (12/115.0)		<.001	1.12 (12/115.0)			.35	
	Treatment	0.47 (1/10.0)		.51		0.09 (1/10.3)		.76	0.25 (1/10.0)			.63	
	Day × Treatment	3.52 (12/115.0)		<.001		5.74 (12/115.0)		<.001	0.44 (12/115.0)			.94	
**Asleep**													
	Day	11.94 (12/91.3)		<.001		10.67 (12/91.3)		<.001	3.70 (12/91.3)			<.001	
	Treatment	1.27 (1/10.0)		.29		0.50 (1/10.0)		.50	0.03 (1/10.0)			.87	
	Day × Treatment	10.20 (12/91.3)		<.001		11.38 (12/91.3)		<.001	4.84 (12/91.3)			<.001	

^a^Df1/df2 indicate the numerator/denominator degrees of freedom.

**Table 4:** Post hoc comparisons between postdose and averaged predose heart rates (mean difference ± SE). The *P* values after Bonferroni correction are provided.

			Asleep													Awake						
Day			Low		*P* value		Median		*P* value		High		*P* value		Low			*P* value		Median		*P* value
**Clenbuterol**																						
	0	3.79 (1.34)		.04		3.7 (1.43)		.08		4.21 (2.42)		.60		–0.68 (2.26)			.99		1.79 (1.92)		.99	
	1	6.54 (1.25)		<.001		7.90 (1.33)		<.001		9.50 (2.26)		<.001		5.30 (2.26)			.15		5.10 (1.92)		.06	
	2	11.60 (1.18		<.001		11.87 (1.26)		<.001		14.57 (2.13)		<.001		8.79 (2.26)			.001		10.79 (1.92)		<.001	
	3	13.88 (1.18)		<.001		16.18 (1.26)		<.001		15.47 (2.13)		<.001		13.64 (2.26)			<.001		14.54 (1.92)		<.001	
	4	13.49 (1.34)		<.001		13.45 (1.43)		<.001		12.23 (2.42)		<.001		15.01 (2.26)			<.001		16.35 (1.92)		<.001	
	5	14.77 (1.34)		<.001		15.48 (1.43)		<.001		14.62 (2.43)		<.001		10.18 (2.26)			<.001		11.41 (1.92)		<.001	
	6	13.85 (1.25)		<.001		15.18 (1.33)		<.001		14.43 (2.26)		<.001		10.16 (2.26)			<.001		12.22 (1.92)		<.001	
**Placebo**																						
	0	–0.66 (1.86)		.99		–1.27 (1.99)		.99		1.08 (3.37)		.99		–0.61 (3.15)			.99		–4.23 (2.67)		.82	
	1	2.27 (1.86)		.99		2.06 (1.99)		.99		2.77 (3.37)		.99		–1.45 (3.15)			.99		–2.48 (2.67)		.99	
	2	1.23 (1.86)		.99		1.23 (1.99)		.99		–2.93 (3.37)		.99		3.90 (3.15)			.99		2.02 (2.67)		.99	
	3	–0.46 (1.92)		.99		–0.77 (2.05)		.99		–4.80 (3.48)		.99		–0.76 (3.15)			.99		–0.48 (2.67)		.99	
	4	0.16 (1.67)		.99		–0.28 (1.78)		.99		–0.82 (3.02)		.99		–0.88 (3.15)			.99		0.02 (2.67)		.99	
	5	0.51 (1.86)		.99		–0.44 (1.99)		.99		–0.81 (3.37)		.99		–3.86 (3.15)			.99		–4.23 (2.67)		.82	
	6	1.17 (1.86)		.99		–0.94 (1.99)		.99		4.68 (3.37)		.99		0.14 (3.15)			.99		–3.98 (2.67)		.98	

**Table 5:** Cohen *d* effect sizes to detect clenbuterol treatment.

	Asleep, *d* effect size				Awake, *d* effect size		
Day	Low	Median	High	Low		Median	High
0	0.73	0.63	0.49	–0.08		0.21	–0.20
1	1.25	1.34	1.11	0.64		0.60	0.68
2	2.22	2.01	1.70	1.06		1.28	0.72
3	2.66	2.75	1.80	1.64		1.72	0.42
4	2.58	2.28	1.42	1.81		1.94	0.66
5	2.83	2.63	1.70	1.22		1.35	0.11
6	2.65	2.58	1.68	1.22		1.45	0.65

## Discussion

### Principal Findings

HR estimates from wearable PPG sensors have been demonstrated to correlate well with the actual HR [8,10]. Here, we further explored the feasibility of using a smartwatch-based PPG HR monitor in clinical trials by assessing the repeatability and sensitivity of HR estimates. All data presented here were part of a randomized placebo-controlled trial including patients with Parkinson disease. Patients wore a smartwatch for a total duration of 13 days. HR estimates were separated into asleep and awake periods based on the smartwatch provided sleep states After the first 6 days, patients started taking daily doses of either a placebo or clenbuterol. Clenbuterol is a β_2_-adrenoceptor agonist known to increase HR [12]. Clenbuterol has been used clinically for decades and has a relatively benign safety profile. The main effects are cardiovascular, with acute increases in HR, increases in systolic blood pressure, and decreases in diastolic blood pressure. Studies in patients with reversible obstructive airways disease treated with clenbuterol have demonstrated statistically significant increases in HR [13], and case reports in humans mention tachycardia as an adverse effect [13-15].

While the smartwatch estimated sleep and awake stages, it is not clear how exactly the algorithm derives these states. The sleep duration can be estimated reasonably well using consumer smartwatches in healthy participants [11]. However, it is unknown how well this algorithm performs in a patient population and under treatment conditions. As the HR and movement is affected by clenbuterol, this could also act as a confounding parameter in the sleep state algorithm. We found, however, that the sleep duration was estimated with moderate repeatability (ICC=0.64) and was not significantly affected by the treatment. Additionally, the number of missing nights was similar both prior to treatment and during treatment. Therefore, we argue that the sleep stage estimates are fit for our purpose.

The smartwatch HR estimates were also found to be moderately repeatable, but most repeatable when patients were asleep (Table 2). The lower repeatability values for awake HR measures can be explained by the larger within-subject variability during the day. Other research groups also found that HR measures at rest are more repeatable than when active [10]. In addition to the higher repeatability values, asleep HR measures had lower MDE values suggesting higher sensitivity while patients were asleep than while patients were awake. Therefore, HR measures while asleep can be considered more repeatable and are potentially more sensitive to detecting changes in HR than HR measures while awake.

Both awake and asleep HR measures were able to detect the effect of clenbuterol (Table 3). In line with the MDE values, the asleep HR measures showed higher sensitivity than the awake HR measures. The increase in HR due to clenbuterol was detected on the first day after dosing using the asleep HR measures but was detected only after the first postdose day using the awake HR measures (Table 4). Additionally, the Awake-High HR marker was unable to detect a change at all due to the large within-subject and between-subject variability (Figure 2). On the other hand, the Asleep-Low HR marker showed the highest sensitivity. All in all, the asleep HR measures are more likely to detect medication effects than awake HR measures.

Drug development trials are generally expensive [15], especially when increasing the number of participants and trial duration (eg, phase 2 or 3 trials). Continuous HR and sleep state values are typically unavailable in clinical trials, especially when subjects are ambulant. However, smartwatches can easily be integrated in such trials for continuous monitoring of participants who go about their daily routines. Additionally, combining a smartwatch with a remote monitoring app installed on a mobile phone (eg, to track mobile phone sensor data) allows passive tracking of both social and physical activities. It should be noted, however, that many smartwatches are not marked as a medical device and therefore cannot be used for diagnostic or treatment purposes. Regardless, smartwatches can detect treatment-induced changes in the HR.

### Limitations

A limitation of this study is that although the asleep HR measures showed higher sensitivity to clenbuterol-induced changes than the awake HR measures, the number of missing data points was substantially higher during the night than during the day. The data loss might be explained by the following: (1) PD-related sleep disturbances [16], (2) reduced compliance with the study protocol, or (3) the use of estimated sleep states provided by the smartwatch. Patients might find the smartwatch unpleasant to wear during the night and decide to either loosen the strap or remove the watch from the wrist. Additionally, at least 1 patient mentioned having trouble falling asleep and being restless throughout the night. This could result in either sleepless nights or inaccurate estimates of sleep states. Instead of relying on estimated sleep states, a fixed time window could be used (eg, between 11 PM and 6 AM).

Another limitation is the arbitrary choice for the quantification of the HR time series. Although we demonstrate that these are sensitive to treatment-induced changes in the HR, other percentiles could have been chosen as well. More sophisticated time series analysis could be used to describe the HR patterns (eg, nonlinear regression analysis). The second arbitrary choice we made in this study is that we assumed patients to be awake between 9 AM and 9 PM when sleep states were missing during the night. Such a choice can be improved by, for example, adding a pedometer or other sensor to detect steps or movements.

### Conclusion

We demonstrated the feasibility of using smartwatch-based HR measurement in early phase clinical trials. The smartwatch was able to detect drug-induced changes in the HR without the need for patients to be present in-clinic. Additionally, such devices allow researchers to obtain frequently sampled HR estimates with limited discomfort for the patient. We demonstrated that HR estimates captured while asleep were more repeatable and most sensitive to drug-induced changes than measures taken while awake. Future studies for validation of commercially available devices should include larger sample sizes and could also include other relevant cardiac parameters, such as the HR variability.

## Appendix

Multimedia Appendix 1 CONSORT-eHEALTH checklist (V 1.6.1).

## Abbreviations

ECG: electrocardiogram
HR: heart rate
ICC: intraclass correlation coefficient
LMM: linear mixed model
MDE: minimum detectable effect
PPG: photoplethysmography
